# Telitacicept monotherapy for refractory idiopathic membranous nephropathy: a case report and literature review

**DOI:** 10.3389/fmed.2025.1571616

**Published:** 2025-04-08

**Authors:** Shucheng Chen, Yiqi Huang, Zhongjie Qu

**Affiliations:** ^1^Department of Endocrinology, Shaoxing Second Hospital, Shaoxing, Zhejiang, China; ^2^Department of Nephrology, Shaoxing Second Hospital, Shaoxing, Zhejiang, China; ^3^Department of Nephrology, The Third Affiliated Hospital of Zhejiang Chinese Medical University (Zhongshan Hospital of Zhejiang Province), Hangzhou, Zhejiang, China

**Keywords:** telitacicept, refractory idiopathic membranous nephropathy, PLA2R, case report, literature review

## Abstract

**Background:**

Patients with refractory membranous nephropathy (MN) face risks of progressive renal decline and end-stage renal disease (ESRD), with limited treatment efficacy. Telitacicept, a novel humanized recombinant fusion protein effective in lupus nephritis and immunoglobulin A nephropathy (IgAN), has few reports on its use in refractory MN.

**Case presentation:**

In May 2023, an 82-year-old man was admitted to Shaoxing Second Hospital with bilateral lower extremity edema. A renal biopsy confirmed idiopathic membranous nephropathy (IMN). Standard therapies, including glucocorticoids (GC), cyclophosphamide (CYC), tacrolimus (TAC), and rituximab (RTX), were ineffective. He developed steroid-induced diabetes and acute renal failure during treatment. Complete proteinuria remission was achieved with telitacicept monotherapy. The patient is under ongoing clinical follow-up.

**Conclusion:**

Telitacicept holds promise as a potential second-line therapy for refractory MN when conventional treatments prove ineffective. However, due to the current lack of robust evidence supporting its use in IMN, further research is warranted to establish its clinical efficacy and safety.

## 1 Introduction

Idiopathic membranous nephropathy (IMN) is a common form of nephrotic syndrome (1). Its cause is linked to autoimmune reactions mediated by antibodies against phospholipase A2-receptor (PLA2R) and thrombospondin type I domain-containing 7A (THSD7A) on podocytes (2), which account for about 60% and 5% of IMN antigens (3, 4), respectively. Despite achieving remission in some patients through conventional immunosuppressive therapy, ~20%−30% of cases exhibit refractory membranous nephropathy (MN) characterized by treatment resistance, frequent relapses, or drug intolerance (5). Such patients are at long-term risk of progressive deterioration of renal function and end-stage renal disease. There is an urgent need in clinical practice to explore safer and more precise treatment options. In recent years, targeted therapies for B cell aberrant activation and autoantibody production have become a key research focus. Telitacicept, a novel humanized fusion protein, binds with high affinity to B cell activating factor (BAFF), and a proliferation-inducing ligand (APRIL), inhibiting excessive B cell activation (6, 7). Theoretically, it provides a potential new approach for treating MN. Although telitacicept has shown efficacy in the treatment of autoimmune nephritis such as lupus nephritis (LN) and immunoglobulin A nephropathy (IgAN) (8, 9), the clinical evidence for its use in MN is extremely limited, especially for refractory MN that has failed standard treatment. The effectiveness and safety of telitacicept in this context remain unclear. In this article, we present a case of refractory MN treated with telitacicept alone and review the clinical features and treatment experience of previous cases.

## 2 Case presentation

On May 6, 2023, an 82-year-old male was admitted to the Nephrology Department of Shaoxing Second Hospital with a 3-month history of bilateral lower extremity edema. Upon admission, the physical examination revealed a blood pressure of 138/84 mmHg, a body temperature of 36.8°C, and severe bilateral lower extremity edema. Laboratory examination was as follows: urine protein 4+, Urinary Albumin-to-Creatinine Ratio (UACR) ≥300 mg/g, 24 h urinary protein (24UP) 12.3 g, albumin (ALB) 16 g/L, total cholesterol 8.72 mmol/L, calcium 1.92 mmol/L, serum creatinine (SCR) 66 μmol/L, and hemoglobin (HB) 136 g/L. The antibodies of anti-PLA2R, anti-THSD7A, anti-NELL1, tumor markers, ANA, ANCA, and immunofixation electrophoresis are all negative. An abdominal ultrasound revealed that the kidneys were normal in both size and shape on both sides.

**Figure 1:**
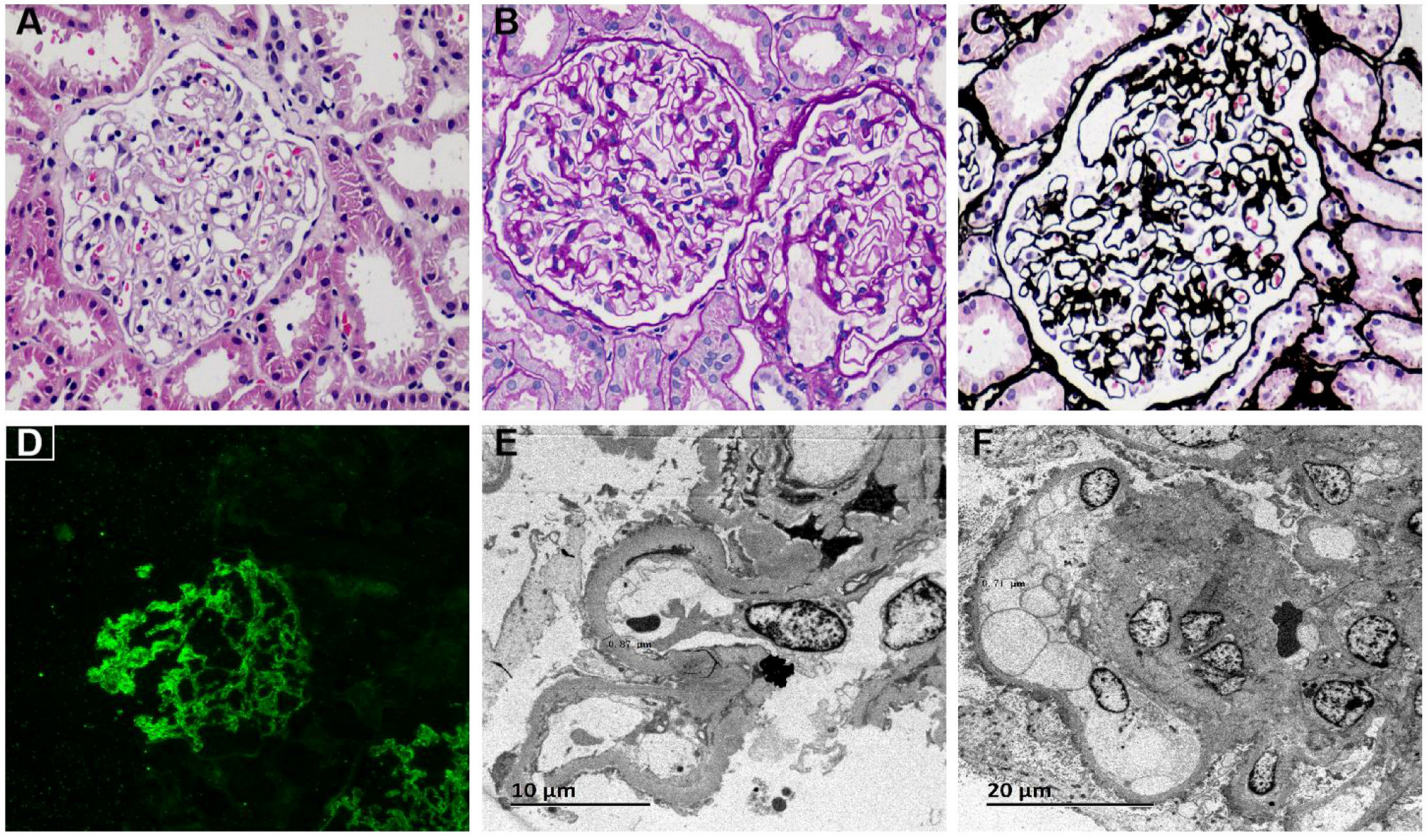
Patients with IMN showed glomeruli sclerosis, diffusely thickened capillary walls and diffuse ribbon-shaped vacuolar degeneration. **(A)** Hematoxylin–eosin staining showed diffuse thickening of the glomerular capillary walls (×400); **(B)** Periodic acid Schiff staining showed granular/vacuolar degeneration of renal tubular epithelial cells (×400); **(C)** Periodic acid silver methenamine staining showed diffuse “satin-like” and vacuolar degeneration of the glomerular basement membrane (×400); **(D)** Immunofluorescence staining showed diffuse fine granular PLA2R deposition along the capillary walls. (×200); **(E)** Electron microscopy showed scattered electron-dense deposits beneath the capillary basement membrane epithelium (×2,500), bar = 10 μm; **(F)** Electron microscopy showed scattered electron-dense deposits beneath the capillary basement membrane epithelium (×1,500), bar = 20 μm.

The patient was initially diagnosed with NS and subsequently underwent a renal biopsy on May 9, 2023. Light microscopy revealed 17 glomeruli, with one showing global sclerosis and the rest having diffusely thickened capillary walls. PASM staining showed diffuse ribbon-shaped vacuolar degeneration of the glomerular basement membrane, with “spikes” in some glomeruli. Visceral epithelial cells were swollen, and the glomerular capsule wall exhibited segmental thickening and partial layering. Renal tubular epithelial cells showed granular and vacuolar degeneration, occasional protein casts, and occasional tubular atrophy. Immunofluorescence results are as follows: IgG 3+, IgA negative, IgM negative, C3 trace, C1q negative, Kappa 3+, Lambda 3+, IgG1 2+, IgG2 negative, IgG3 negative, IgG4 2+, PLA2R +, THSD7A–. Electron microscopy revealed scattered electron-dense deposits subepithelially in most segments of the glomerular capillary basement membrane. In a few segments, string-of-beads-like electron-dense deposits and hyperplasia of the basement membrane between these deposits, forming “spike-like” structures, were observed. The thickness of the basement membrane ranged from 310 nm to 1300 nm, with diffuse foot process fusion present. Based on the clinical data and findings from light microscopy, electron microscopy, and immunofluorescence examination, the pathological diagnosis is MN stage I-II (Figure 1).

Based on the risk stratification for MN outlined in the Kidney Disease: Improving Global Outcomes (KDIGO) 2021 Clinical Practice Guideline on Glomerular Diseases, the patient was assessed to have a high risk of progressive renal function decline. Consequently, the patient received the standard treatment regimen of prednisone (30 mg po qd) and cyclophosphamide (CYC) (1 g IV on May 15, June 14, July 14, August 15, September 17, and October 16, respectively). On November 25, 2023, the patient had a 24UP quantification of 7.57 g and UACR ≥300 mg/g. Given the suboptimal response to CYC therapy, the treatment regimen was adjusted to tacrolimus (TAC) (1 mg po bid) and prednisone (30 mg po qd). Four weeks later, the blood concentrations of TAC was 3.2 ng/mL, with a persistent 24UP level of 6.12 g. Additionally, ALB was 21 g/L, and SCR was 81 μmol/L and UACR was ≥300 mg/g. Based on these findings, the treatment regimen was further modified to TAC (2.5 mg po bid) and prednisone (30 mg po qd).

On February 2, 2024, this patient was admitted to our hospital with a 2-day history of nausea and vomiting. Laboratory findings were as follows: urine protein 3+, blood glucose 29.9 mmol/L, blood pH 7.22, SCR 141 μmol/L, glycated hemoglobin (HbA1c) 11.5%, 24UP 6.16 g, UACR ≥ 300 mg/g, ALB 22 g/L, and TAC concentration 8.2 ng/mL. Oral glucose tolerance test (OGTT) results included: fasting blood glucose 11.5 mmol/L, 0.5 h postprandial glucose 15.5 mmol/L, 1 h postprandial glucose 18.3 mmol/L, 2 h postprandial glucose 14.2 mmol/L, and 3 h postprandial glucose 12.1 mmol/L. The patient was diagnosed with steroid-induced diabetes mellitus, diabetic ketoacidosis, and acute renal failure. Therapeutically, prednisone and TAC were immediately discontinued, and the patient was transitioned to symptomatic and supportive care. We have analyzed that the patient currently presents with refractory MN and steroid-induced diabetes mellitus. Despite standard treatment with CYC and TAC, remission has not been achieved. We recommend initiating a rituximab (RTX) regimen for further management. On February 17, 2024 and March 2, 2024, the patient received intravenous infusions of 1 g rtx, respectively. Before the first infusion, the 24UP was 5.31 g, UACR was ≥300 mg/g, SCR was 125 μmol/L, ALB was 21.9 g/L, and total B cells (CD20+) were 17.5%. Before the second infusion, the 24UP was 5.11 g, UACR was ≥300 mg/g, SCR was 89 μmol/L, ALB was 23.1 g/L, and CD20+ was 2.1%. On March 15, CD20+ was rechecked and found to be 0%. On April 13, 2024, the 24UP was 4.21 g, UACR was ≥300 mg/g, ALB was 25.2 g/L, and CD20+ was 0%. On June 20, 2024, CD20+ was rechecked and found to be 5.2%, the 24UP was 4.10 g, UACR was ≥300 mg/g and ALB was 25.3 g/L. Another 1 g rtx was infused. On August 10, 2024, the 24UP was 4.57 g and CD20+ was 0%.

**Figure 2:**
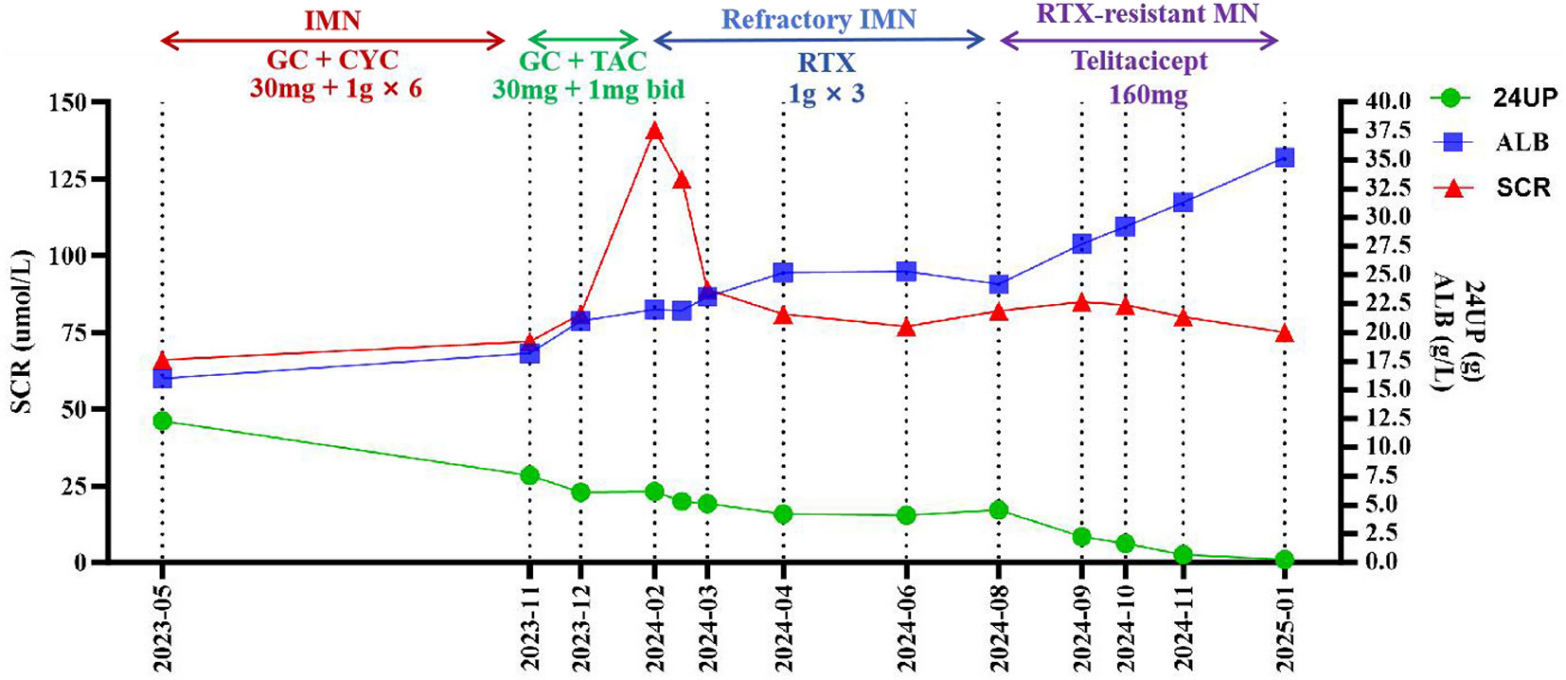
The timeline for diagnosis and treatment.

Given the suboptimal therapeutic response to RTX, the patient was diagnosed with RTX-resistant MN. A comprehensive review of the literature revealed successful cases of treating refractory MN with telitacicept. Following detailed discussions with the patient and their family, it was decided to initiate telitacicept subcutaneous injections at a dose of 160 mg once weekly, starting on August 15, 2024. On September 25, 2024, the 24UP was 2.24 g and UACR was ≥300 mg/g. On October 19, 2024, the 24UP decreased to 1.67 g and UACR was 150 mg/g. Due to the patient's advanced age, telitacicept was reduced to 80 mg subcutaneously weekly. By November 20, 2024, the 24UP was 0.67 g and UACR was 30 mg/g, and serum albumin was 31.3 g/L. Telitacicept was further reduced to 80 mg every 2 weeks. By January 15, 2025, the 24UP was 0.26 g, UACR was < 30 mg/g and serum albumin increased to 35.2 g/L. At this point, the patient has achieved successful remission of membranous nephropathy, leading to the discontinuation of telitacicept therapy. Currently, the patient is still under clinical follow-up. Figure 2 illustrates the timeline for diagnosis and treatment.

## 3 Discussion and conclusion

MN is a prevalent glomerular disease characterized by the deposition of immune complexes on the subepithelial side of the glomerular basement membrane (GBM), leading to diffuse thickening of the GBM. The peak incidence occurs in individuals aged 40 to 60 years, with a male-to-female ratio of ~2:1 (10). Approximately 70% of MN cases are IMN, where the etiology remains unknown. Studies show that IMN prognosis varies widely: about 30% achieve spontaneous remission, and 30–40% progress to end-stage renal disease (ESRD) within 5–15 years (11, 12). In recent years, the management of IMN has primarily adhered to the KDIGO 2021 Clinical Practice Guideline for Glomerulonephritis. For patients with medium-to-high-risk IMN, recommended treatment regimens include glucocorticoids (GC) combined with alkylating agents, GC combined with calcineurin inhibitors, or RTX, achieving a remission rate of ~70%. MN that remains unresponsive to first-line immunosuppressive therapy administered at adequate doses and durations is classified as refractory MN. Given the immunological mechanisms of IMN, blocking key targets can yield beneficial outcomes. Studies have focused on exploring targeted biological agents that effectively block the mechanism of IMN to achieve the best therapeutic effect, including anti-CD20 monoclonal antibodies, B-cell activator inhibitors, plasma cell inhibitors, and complement inhibitors (13). Current research on therapeutic targets for MN is still in the exploratory stage. Miao et al. (14) demonstrated via cell experiments that Sirt6 is a specific target for treating podocyte injury-related kidney diseases like IMN, which reduces proteinuria by blocking RAS signaling through the Wnt1/β-catenin pathway.

Based on its pathological mechanism, MN is considered an autoimmune kidney disease. To date, there is no established consensus on the standard treatment for refractory MN. This report describes a case of successful management of refractory MN using Telitacicept, providing valuable insights for clinical practice. To our knowledge, only five cases of refractory MN treated with telitacicept have been comprehensively reported in the literature to date, all originating from China (15–17). Table 1 summarizes the clinical data of Telitacicept treatment in refractory MN patients. Of the five cases, four were male, with ages ranging from 32 to 79 years. Existing literature indicates that gender and age do not significantly influence the prognosis or treatment response in IMN patients (18). All five cases were IMN, with four at pathological stages I-II. All patients had negative serum anti-PLA2R antibodies, but three showed positive PLA2R by immunofluorescence staining. Anti-PLA2R antibody is a biomarker for diagnosing and predicting IMN, with sensitivity of 40–83.9% and specificity of 89–100% (4, 19, 20). Vellakampadi et al. (21) found that PLA2R immunohistochemical staining has higher sensitivity than serological testing, consistent with our retrospective findings. This patient's renal biopsy showed positive PLA2R antigen but negative serum anti-PLA2R antibody. This does not necessarily indicate IMN with negative serum anti-PLA2R antibody. Possible explanations include: (1) The patient may be in an immune quiescent state, with residual PLA2R antigen deposition from prior immune responses (22). (2) Serum anti-PLA2R antibody levels might be below the detection threshold, yet local renal antigen deposition persists. Therefore, we recommend combining serum anti-PLA2R antibody testing with PLA2R immunohistochemical staining for diagnosing IMN in patients with shorter disease duration. Unfortunately, in this case, serum anti-PLA2R antibody were only tested at the initial visit and not re-evaluated during RTX and telitacicept treatment.

**Table 1:** Case summary of telitacicept in the treatment of MN.

**References**	**Country**	**Gender**	**Age**	**Serum anti-PLA2R antibody**	**Immunofluorescence staining of PLA2R**	**Pathological staging of MN**	**Previous treatment options**							**Current therapeutic options**	**Outcome**
							**GC**	**TAC**	**MMF**	**MZR**	**CYA**	**CYC**	**RTX**		
Wang et al. (17)	China	Male	32	Negative	Positive	Stage I-II		A					B	Telitacicept (160 mg qw)	Partial remission
Zhang et al. (15)	China	Male	46	Negative	Positive	Stage I-II	ABCD	A	B			C	D	GC combined with telitacicept (160 mg qw)	Full remission
Sun et al. case 1 (16)	China	Male	40	Negative	Positive	Stage III	AB				A		B	GC combined with telitacicept (240 mg qw)	Full remission
Sun et al. case 2 (16)	China	Female	63	Negative	Negative	Stage I-II	AB	A		A		B		GC combined with telitacicept (240 mg qw)	Partial remission
Sun et al. case 3 (16)	China	Male	79	Negative	Negative	Stage I-II							A	Telitacicept (240 mg qw)	Non-remission
Present report (2025)	China	Male	82	Negative	Positive	Stage I-II	AB	B				A	C	Telitacicept (160 mg qw)	Full remission

A, First treatment option; B, Second treatment option; C, Third treatment option; D, Fourth treatment option; MZR, Mizoribine; CYA, Cyclosporine A.

Selecting and adjusting treatment regimens for IMN is a major clinical challenge. The 2021 KDIGO guideline classifies IMN patients into low, moderate, high, and very high-risk groups and recommends immunosuppressive therapy for those in moderate, high, and very high risk categories (23). In our review, 3 out of 5 cases used traditional regimens combining GC with immunosuppressant's. The 2021 KDIGO guidelines recommend GC plus Calcineurin Inhibitors (CNI) for moderate risk IMN patients and GC plus CYC, CNI, or RTX for high risk patients (23). A systematic review analyzing 22 studies encompassing 1,971 IMN patients highlighted that the combination of GC and CYC exhibited superior long-term efficacy and a significantly lower recurrence rate in IMN treatment (24). GC plus CYC is the only regimen proven to delay ESRD progression but has a higher risk of adverse events (25). GC plus CNI shows similar efficacy to alkylating agents but has a 50% relapse rate after discontinuation. GC plus mycophenolate mofetil (MMF) is not KDIGO's first choice but may reduce steroid exposure, as proposed by Sharma et al. (26), though evidence is limited. As shown in Table 1, four out of five patients, including our case, switched to RTX after immunosuppressive therapy failed. Studies suggest that RTX may provide better outcomes for IMN patients unresponsive to conventional treatments. However, about one-third of MN patients do not respond to RTX (27). In this case, this patient also showed resistance to RTX treatment, which may be related to multiple mechanisms. Firstly, studies have shown that the resistance of IMN may be associated with different subgroups of B cells. RTX depletes B cells by targeting CD20, but some B cell subgroups, such as CD38+/CD138+ long-lived memory plasma cells, may not be affected by RTX (28). These cells can continuously produce autoantibodies, leading to the persistence and recurrence of IMN. Secondly, research also indicates that the resistance of IMN may be related to the activation of components of the immune system such as T cells and the complement system. RTX mainly targets B cells, but the activation of T cells and the complement system may also play a role in RTX resistance (29). Finally, individual differences may also be an important factor leading to RTX resistance. The genetic background, disease severity, and drug metabolism capacity of different patients may result in different responses to the efficacy of RTX (30). In addition, optimizing RTX dosage and treatment duration to improve the prognosis of IMN is currently a research focus. A single-center retrospective study of 41 IMN patients found that the new regimen (100 mg RTX for 6 months) achieved similar CD20+ B cell depletion as the standard regimen, with significantly lower cumulative dose and safety risks (31). Notably, Wang et al. (17) chose TAC monotherapy without GC, likely due to the patient's long-term immune deficiency from HIV and tuberculosis. Furthermore, Chinese herbal medicine (CHM) has demonstrated promising efficacy in treating IMN. A prospective, multicenter cohort study including 2,000 adult IMN patients reported a combined remission rate of 67.0% with CHM monotherapy or combination therapy (32). A meta-analysis of 30 randomized controlled trials (RCTs) indicated that all treatment regimens involving tripterygium glycosides were more effective than GG; among the monotherapy regimens for IMN, tripterygium glycosides were comparable to CNI and significantly better than GC (33). A network meta-analysis of 31 RCTs involving 2,195 IMN patients and 15 different CHMs indicated that integrating CHM with biomedicine could provide substantial benefits, particularly in reducing 24UP levels and enhancing serum albumin concentrations (34).

All five cases were treated with telitacicept, and three of them also received GC therapy. For this case, considering the patient's advanced age and the coexistence of steroid-induced diabetes, telitacicept monotherapy was chosen. Four out of five patients achieved proteinuria remission after telitacicept treatment: two had complete remission, and two had partial remission. Only the case reported by Wang et al. (17) did not show remission. However, from the clinical indicators of this patient, although NS persisted, the indicators such as proteinuria, renal function, and serum albumin all improved compared to before treatment. We suggest that the patient can continue to use telitacicept and combine it with a low dose of GC, which may have a positive effect on the outcome. In our case, telitacicept monotherapy led to complete remission of nephrotic syndrome, a very encouraging result.

Currently, telitacicept has been recommended for the treatment of autoimmune nephritis such as LN and IgAN (35–37). Neusser et al. (37) found that the mRNA levels of APRIL and BAFF in the glomeruli of patients with proliferative LN were significantly increased, and the expression of APRIL, BAFF, and B-cell maturation antigen in plasma also significantly increased. As a decoy receptor, tectacicept can effectively inhibit the overactive B-cell signaling (23). A single-center retrospective study of 30 LN patients with inadequate GC response showed a 73.3% complete remission rate with telitacicept, reducing the doses of glucocorticoids and immunosuppressants while maintaining good safety (36). Li et al. (38) found that the serum levels of galactose-deficient (Gd)-IgA1 and the density of IgA deposition in glomerular mesangium in patients with IgAN were positively correlated with serum BAFF, while the levels of Gd-IgA1 and the severity of IgAN were positively correlated with APRIL levels. Further study confirmed that BAFF and APRIL might be the inducements for the abnormal production and secretion of Gd-IgA1 (39). Therefore, targeting BAFF/APRIL may become a new strategy for the treatment of IgAN. A single-center, three-arm, open-label study of 62 IgAN patients showed that median proteinuria in the telitacicept group decreased by 54.6% from baseline, compared to 20% in the supportive treatment group and 72.1% in the immunosuppressive treatment group (35). The eGFR in the telitacicept group increased by 4.3%, while it decreased by 5.8% and 8.4% in the other two groups (35). These results suggest that telitacicept effectively reduces proteinuria and stabilizes eGFR in IgAN patients.

The pathological mechanism of IMN is complex, involving oxidative stress and inflammatory response. Excessive ROS or weak antioxidant defense causes oxidative stress, damaging the glomerular basement membrane (40), upregulating PLA2R expression, and promoting IMN progression (41). Secondly, inflammatory response is a key feature of IMN. The release of inflammatory factors can lead to glomerular cell damage and fibrosis, thereby aggravating the condition. Studies have shown that inhibiting inflammatory signaling pathways such as NF-κB can effectively reduce the inflammatory response and damage in the kidneys (42). BAFF-R is a type III transmembrane protein expressed in B cells (43). After binding to its unique ligand BAFF, it promotes the survival of mature B cells by activating both canonical and non-canonical NF-κB pathways (44). NF-κB regulates the expression of BAFF-R by binding to an NF-κB binding site in the BAFF-R promoter, indicating that inhibiting NF-κB can reduce the expression of BAFF-R gene and protein and enhance the activity of the BAFF-R gene (44). Therefore, the NF-κB binding site in BAFF-R may become a new therapeutic target for IMN. Telitacicept leverages the high affinity of the TACI receptor for BAFF and APRIL ligands to inhibit their interaction with cell membrane receptors, including BAFF-R (6). This mechanism effectively blocks the suppression of B lymphocyte proliferation and T lymphocyte maturation. Given the pathogenesis of IMN and telitacicept's target, it is reasonable to expect telitacicept to have broad prospects in treating refractory MN.

## 4 Conclusion

Overall, we reported a case of successful treatment of refractory MN with telitacicept. We believe that telitacicept is expected to become a new second-line therapy for refractory IMN when standard treatments fail. However, more large-scale, prospective studies are needed to confirm its clinical efficacy and safety, as current evidence is limited.

## Data Availability

The original contributions presented in the study are included in the article/supplementary material, further inquiries can be directed to the corresponding author.
